# Direct and Rapid Identification of Vibrio Cholerae Serogroup and Toxigenicity by a Novel Multiplex Real-Time Assay

**DOI:** 10.3390/pathogens11080865

**Published:** 2022-07-30

**Authors:** Yong Yan, Li Zhan, Guoying Zhu, Junyan Zhang, Ping Li, Lixia Chen, Peiyan He, Jianyong Luo, Zhongwen Chen

**Affiliations:** 1 Jiaxing Key Laboratory of Pathogenic Microbiology, Jiaxing Municipal Center for Disease Control and Prevention, Wenqiao Road 486, Jiaxing 314050, China; 13567309672@139.com (Y.Y.); jxcdczhuguoying@163.com (G.Z.); ahliliping@163.com (P.L.); clx9685@163.com (L.C.); hepeiyan@aliyun.com (P.H.); luojy10@163.com (J.L.); 2 Institute of Microbiology, Zhejiang Provincial Center for Disease Control and Prevention, Binsheng Road 3399, Hangzhou 310051, China; lzhan@cdc.zj.cn (L.Z.); jyzhang@cdc.zj.cn (J.Z.)

**Keywords:** *Vibrio cholerae*, cholera toxin, direct PCR, multiplex real-time PCR, molecular diagnosis

## Abstract

Molecular diagnostic assays for cholera detection have superior sensitivity to conventional assays and are now being accepted as the new standard method, especially the real-time PCR/RT-PCR. However, limited throughput capacity and long detection duration prevent them from detecting more specimens and more targets in one turnaround time simultaneously. In this study, we utilized nucleic acid extraction-free, direct RT-PCR and high-speed amplification to develop a novel multiplex assay, a quadplex direct one-tube real-time RT-PCR assay, for rapid detection of the serogroup and cholera toxin toxigenicity of *Vibrio cholerae* targeting the *epsM*, *ctxA*, *rfb-O1*, and *rfb-O139* genes. Performance of the multiplex assay was evaluated by comparison with the monoplex real-time PCR assay according to the China Cholera Prevention Manual. Detection data from clinical specimens showed that the new assay had good diagnostic sensitivities for *epsM* (100%, n = 301), *ctxA* (100%, n = 125), *rfb-O1* (100%, n = 85), and *rfb-O139* (97.87%, n = 49). Analysis of the analytical sensitivities with serial dilutions of positive standards showed that the detection limits of the new assay for *Vibrio cholerae* *epsM,* *ctxA,* *rfb-O1*, and *rfb-O139* were up to 200, 590, 115, and 1052 copies per mL lower than the monoplex real-time PCR (910, 345, and 1616 copies/mL respectively, for *ctxA,* *rfb-O1*, and *rfb-O139*). The results indicate that the multiplex assay is a rapid, sensitive, specific, and easy-to-use detection tool for *Vibrio cholerae*, especially suitable for rapid identification and screening detection of mass specimens.

## 1. Introduction

Cholera is a worldwide epidemic and older infectious disease. Since 1817, from its original reservoir in the Ganges delta in India, there have been seven worldwide pandemics. The past six were caused by *Vibrio cholerae serogroup* O1 biotype classical, and the current pandemic was caused by *V. cholerae* serogroup O1 biotype El Tor and spread across all continents [1,2,3]. The serogroup O139 of *V. cholerae* was found in Bangladesh in 1992 and caused some outbreaks in the past, but it has never being identified outside Asia [4]. There was no difference in the illness caused by the two serogroups due to the same virulence, mainly cholera toxin (CT) and similar encoding genes [4,5,6]. In recent years, the cholera toxin (CT)-producing or cholera toxin gene (*ctx*)-positive strains of *V. cholerae* non-O1/non-O139 serogroups have been extensively reported [7,8,9], as their pathogenicity and potential harm are worthy of attention. Diagnostic serums are often used to rapidly identify *V. cholerae* strains; however, it is sometimes difficult to distinguish them easily using the serum agglutination test alone, especially between the serotypes in the O1 serogroup.

At present, there are three main types of detection methods for *V. cholerae*: (1) Traditional isolation culture and identification methods, including pathogen isolation culture, serological diagnosis, biochemical identification, and phage lysis test. The whole detection takes a long time (the fastest takes 3 days), the operation is complicated, and the reagent consumption is large. (2) Time-of-flight mass spectrometry identification can analyze bacteria-specific proteins within a short time, only about ten minutes, but it requires isolating culture to obtain bacterial monoclonal strains. The entire detection process generally takes more than 24 h and can only reach the species level of *V. cholerae*, and the serogroup and toxigenicity cannot be identified. Especially, the instrument is expensive. (3) Molecular biological methods based on nucleic acid are popular and the use of PCR and real-time PCR instruments can shorten the detection time of *V. cholerae* to 6 h (real-time PCR can shorten it to <3 h [10,11,12,13]), but the problems faced are: inconsistent methods, many reports, and different target genes for research, so that the specificity and sensitivity are different [10,11,12,13,14,15]. Since the *Cholera Prevention Manual* (Sixth Edition) was issued by the Ministry of Health of China in 2013, the real-time PCR method [16] has been adopted by most grassroot units in China, but it is a single-target analysis and cannot identify non-O1/non-O139 serogroup strains, so the routine detection of *V. cholerae* species still depends on traditional isolation and identification methods, which still cannot meet the needs of cholera prevention and control for simpleness, precision, and high efficiency. Therefore, more rapid, specific, simple, and easy-to-promote technologies and methods undoubtedly have broad application spaces.

In this study, one-step multiplex reverse-transcription-PCR (RT-PCR), high-speed amplification, and direct PCR technology were utilized to develop a novel multiplex real-time RT-PCR assay for simultaneous detection of four targets, *epsM*, a species-specific gene in all strains of *V. cholerae*; *ctxA* encoding cholera toxin; and *rfb-O1* and *rfb-O139* encoding the O1 and O139 lipopolysaccharide antigens. Due to the use of the *epsM* gene in combination with *rfb-O1* and *rfb-O139*, this assay could not identify *V. cholerae* O1 and O139 as causing the cholera epidemic but could identify *V. cholerae* non-O1/non-O139 serogroups, which are not rare in localized diarrhea. In addition, the assay had the characteristics of nucleic acid extraction-free, one-tube detection, and a shorter detection time, which made it faster and easier to use. The performance of this new assay was evaluated by comparing it with a previous monoplex real-time PCR assay based on the *Cholera Prevention Manual*, using practical specimens, isolated strains, and synthetical plasmids from target sequences of *V. cholerae*.

## 2. Results

### 2.1. Analysis of Analytical Performance

**Figure 1:**
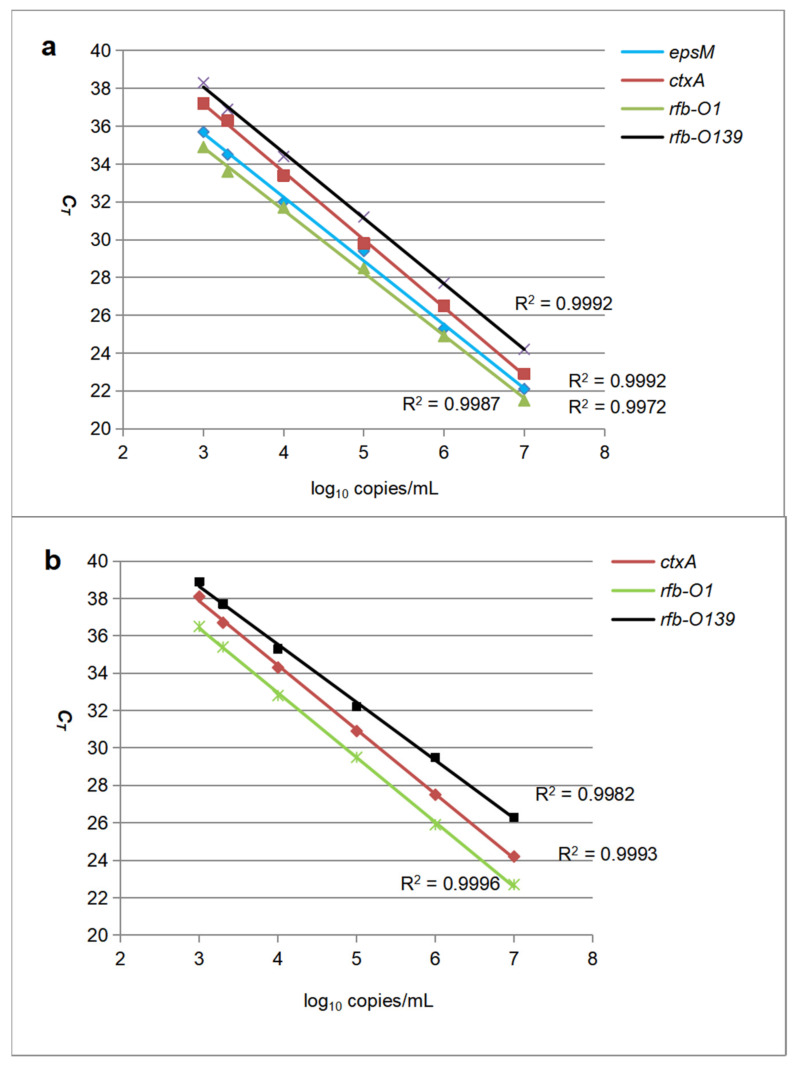
Comparison of the analytical sensitivity of the new multiplex real-time assay (**a**) and previous monoplex real-time assay (**b**). Bacterial in vitro DNA plasmids of four targets were used as standards and the concentrations of each target in the standard curves were expressed in log_10_ copies/mL versus threshold cycle values (*C_T_*) for the two assays.

The analytical sensitivities of the previous monoplex real-time PCR and the new multiplex real-time RT-PCR were assessed by testing in duplicate 10-fold serial dilutions of positive standards ranging from 10^6^ to 10^1^ copies/mL of target DNA plasmids for each target. The standard curves were drawn by the log_10_ values of the standard concentrations versus the *C_T_* values for two real-time assays (Figure 1). From calculation using the curves at the pre-set cutoff value mentioned above, the detection limits of the monoplex real-time assay were determined to be 910, 345, and 1616 gene copies per mL (equivalent to 23, 9, and 40 copies per reaction) for *ctxA,* *rfb-O1*, and *rfb-O139*, respectively. The data of the multiplex real-time assay were 200, 590, 115, and 1052 copies per mL (equivalent to 5, 15, 3, and 26 copies per reaction) for *epsM,* *ctxA,* *rfb-O1*, and *rfb-O139*. 

**Figure 2:**
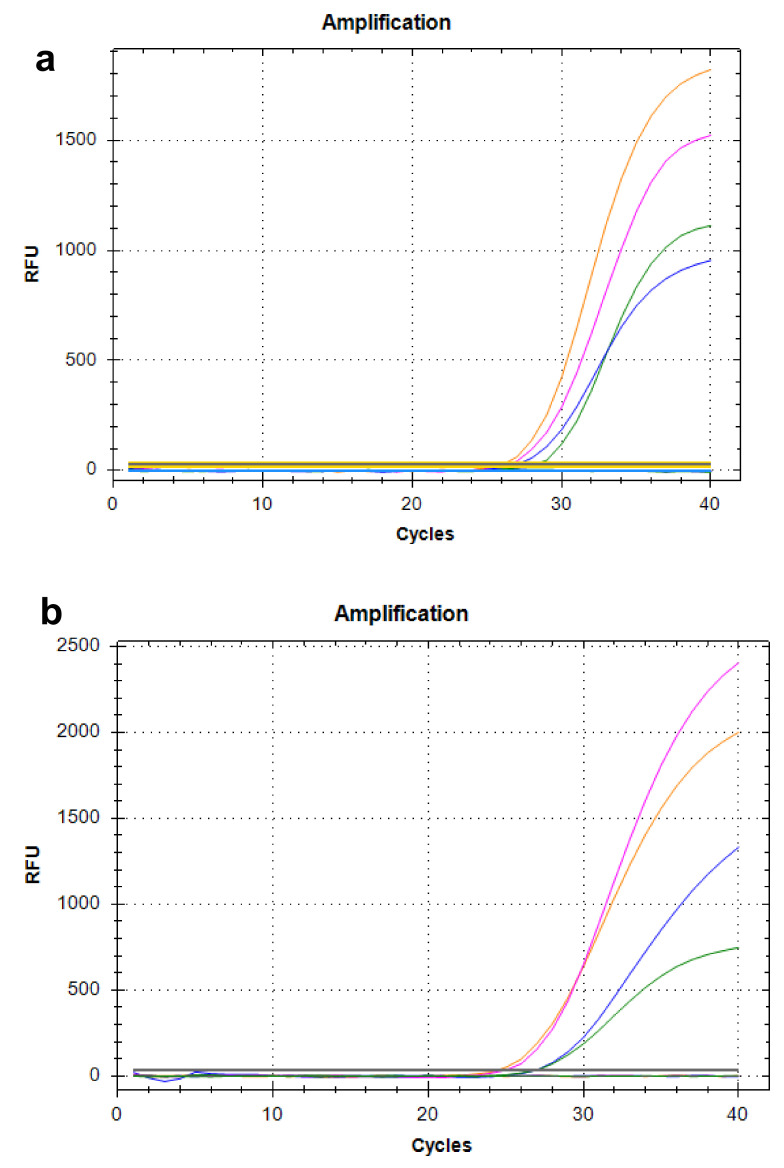
Suitable unified positive control for new multiplex assay to obtain a satisfactory amplification plot. Targets are shown as pink for the *epsM* gene, green for *ctxA*, yellow for *rfb-O1*, and blue for *rfb-O139*. Mix-PC1, the mixture in equal amounts with 10^5^ gene copies per mL of four positive controls (**a**); Mix-PC2, the mixture in equal amounts with 0.5 MCF (McFarland concentration) bacterial suspensions of O1 (No. 130605, *ctx*-positive) and O139 (No. 200513, *ctx*-negative) culture strains (**b**).

The analytical performance of the two assays was also assessed by testing the diagnosed specimens, isolated strains, and positive controls. As shown in Table 1, both assays were able to detect all target bacteria and no cross reactions were observed. In order to further examine the performance of the multiplex assay for mixed infection or complexed specimens, two mixtures, PC-Mix1 and PC-Mix2, containing four targets, respectively, prepared with positive controls and isolated strains, were tested in this study and each target was detected successfully with no interference signals from the others. The results showed that the new multiplex real-time assay had gratifying analytical specificity and multiplex detection capacity for all target genes, and Mix-PC1, the mixture in equal amounts with 10^5^ gene copies per mL of the four positive controls, was more suitable as a unified positive control of the multiplex assay to obtain a satisfactory amplification plot (Figure 2). 

### 2.2. Analysis of Diagnostic Performance 

In total, 338 practical specimens were tested using the monoplex real-time PCR assay and new developed multiplex real-time assay to assess their diagnostic performance in clinical application. The diagnostic sensitivity and specificity of the new multiplex assay for each target gene were analyzed with software SPSS version 19.0, using the results of the monoplex assay as a comparator (Table 2). Due to the lack of the species target gene for *Vibrio cholerae* in the monoplex real-time PCR, *ctxA* was previously used as an alternative to detect *V. cholerae*, especially in epidemic periods of cholera. Results showed that the multiplex assay has good diagnostic sensitivities for *epsM* (100%, n = 301), *ctxA* (100%, n = 125), *rfb-O1* (100%, n = 85), and *rfb-O139* (97.87%, n = 49), and the *Kappa* values of the data for the *ctxA*, *rfb-O1*, and *rfb-O139* genes were more than 0.95, suggesting great consistency in the diagnostic performance of both assays for the three targets. Additionally, we found that the new multiplex assay detected more positive specimens and O1-serogroup strains, with significant values for the *V. cholerae* species-specific *epsM* (χ^2^ = 175, *p* < 0.001) and *rfb-O1* gene (χ^2^ = 4.167, *p* < 0.05), indicating that the new multiplex assay for the two targets had lower detection limits and higher sensitivities than the monoplex assay.

### 2.3. Analysis of Cost-Effectiveness

The reagent cost and turnaround time of the previous monoplex assay and our developed multiplex assay were calculated and are listed in Table 3. The monoplex real-time PCR assay, due to the need for bacterial nucleic acid extraction before target amplification and analysis, required at least 1.4 h (80 min) to test one plate if all three panels of the assay were set up in one 96-well plate (the number of tested specimens was less than 31 because at least 3 wells were needed for the positive, negative, and blank control). The new multiplex assay took at most 40 min to test one plate (up to 93 specimens, 3 wells for the positive, negative, and blank control) due to the lack of an extraction step of nucleic acids before target amplification.

A workload of two plates (i.e., 2 × 96 reactions) were tested to compare the multi-target and throughput capability of the two assays. The previous monoplex assay needed six times to spend up to 5.5 h (330 min) and CNY ¥17,280 (¥90/reaction) for all reactions when only one real-time PCR instrument was available while the new multiplex assay needed at most 1.3 h (80 min) and ¥5760 (¥30/reaction). Within these 6 h, just a 6-h clinical shift, one technician could use the new multiplex assay to test more specimens (4 plates × 93 = 372 specimens) with a real-time PCR instrument. Obviously, it is an easier and more cost-effective alternative to the previous monoplex assay.

## 3. Discussion

The *epsM* gene is a coding gene of exocrine protein system subunit M of *Vibrio cholerae*, which is species-specific [17,18,19]. In this study, it was selected as the target gene to discover all strains of *V. cholerae* regardless of whether they were from the O1\O139 serogroup or the non-O1\non-O139 serogroup. The former, usually an epidemic strain, is of great significance in the cholera epidemic while the latter, as a non-epidemic strain, is often found in localized diarrhea and environmental water [6,15]. It was noteworthy that the CT-producing and *ctx*-positive strains of the *V. cholerae* non-O1/non-O139 serogroups have been increasingly reported [7,8,9] and have also been found in Jiaxing, a water city threatened by cholera in the south of the Yangtze River, China. Therefore, our new developed multiplex assay can not only be used in the emergency detection of *V. cholerae* during an epidemic period but may also play an important role in routine surveillance during non-epidemic periods.

Some commercial kits for *Vibrio cholerae* are available in China at present and some multiplex real-time PCR assays have been reported in previous papers for *ctxA*, *rfb-O1*, *rfb-O139*, and other species-specific genes, such as *hlyA*, *rtxA*, *ompW, rnpB, toxR*, and so on [10,11,12,13,14,15]. However, they either involved complicated steps or included unsatisfactory/unsuitable targets or did not have the species-specific targets needed. Importantly, they were too expensive or/and time-consuming for routine detection in the clinical laboratory. In this study, the reaction system of our new multiplex assay used qRT-PCR reagents with the addition of mild cell-lysing agent and PCR enhancer. The former, Triton X-100, can gently lyse cells without affecting the activity of enzymes during the PCR reaction [20,21], and the latter, Betaine, can facilitate PCR reactions [22,23,24,25], which means that nucleic acid release and detection can be performed simultaneously in one closed tube, and higher sensitivity and specificity can be obtained because both DNA and RNA targets can be detected simultaneously. Since the reagent components, such as reverse transcriptase and DNA polymerase, support high-speed amplification with high tolerance against reaction inhibition, the entire detection time of this assay could be greatly shortened to within 40 min. This is the first report to describe a real one-tube multiplex assay based specifically on real-time RT-PCR, with no nucleic acid extraction, closed-tube detection, and high-speed amplification, for rapid identification of serogroup and CT-producing toxicity of *V. cholerae*.

Over the past ten years, real-time PCR had been adopted by our lab for the diagnosis and surveillance of infectious diarrhea due to the advantage of simultaneous amplification and analysis, without post-amplification manipulation [26,27]. However, all of the primers and probes provided by the *Cholera Prevention Manual* were designed specifically for a single target of *V. cholerae*. Although multiplex real-time PCR was also used in this work, the limited multiplex analysis performance was obvious mainly due to the unoptimized reaction system and adverse interactions between the primers and probes [28,29,30]. So, the previous real-time PCR assay for *V. cholerae* had to be divided into three panels, respectively, for the *ctxA*, *rfb-O1*, and *rfb-O139* genes in this study. This made the limited throughput capacity more detrimental and tighter. In an epidemic period, the monoplex assay would require more plates, more technicians, and increased turnaround time; otherwise, the lab need to buy more machines in parallel to run a large number of samples within a limited work shift. Our study showed that the previous monoplex real-time assay would take four times the time and three times the cost of the new multiplex assay to finish the workload of two plates (2 × 96 reactions).

In view of the facts, the new multiplex assay was developed for rapid-turnaround, multi-target, and high-throughput detection of *Vibrio cholerae*. All of its primes and probes were newly designed and labeled with double black-hole quenching (DBQ) groups for the probes to reduce the interference from background fluorescence [31,32,33,34] This allowed the assay to perform continuous analysis in a plate queue and detect four targets simultaneously with great specificities and sensitivities. Usually, the detection limit of an assay is determined in the following ways: (1) To detect a series of concentrations of standard substances, the lowest concentration at which 95% of the positive samples (at a confidence of 95%) are detected is the limit of detection [35,36]. (2) The corresponding amount of three times the instrumental background signal produced from the blank control (at a signal-to-noise ratio of 3) is the detection limit [37,38,39]. However, real-time PCR has its particularity. Due to the different sensitivities of each target detection, the minimum detection limit of each target of a multiplex real-time PCR assay is different, so the corresponding positive judgment value, that is, the cutoff of *C_T_*, is inconsistent. In addition, when the amplification is late (e.g., *C_T_* over 38), it is prone to non-specific amplification, leading to false positives due to the attenuation of enzyme activity, the consumption of substrates, and the accumulation of non-specific products, such as primer dimers. In fact, a multiplex nucleic acid assay based on real-time PCR usually selects a *C_T_* value corresponding to the highest detection limit among all targets as its uniform cutoff before ensuring the specificity of the reaction (*C_T_* < 40) to ensure that all targets can be detected and facilitate the user to apply this assay. So, in this study, the *C_T_* value of 38 was selected as the cutoff, at which the corresponding values of the new multiplex assay for *epsM*, *ctxA*, *rfb-O1*, and *rfb-O139* were 200, 590, 115, and 1052 copies per mL as their detection limits, and the values of the previous monoplex assay were 910, 345, and 1616 copies per mL for *ctxA, rfb-O1*, and *rfb-O139*, respectively. Statistically, it showed that the diagnostic sensitivity of the new multiplex assay was significantly higher than that of the previous monoplex assay due to the usage of the species-specific target, the *epsM* gene, for *V. cholerae*.

## 4. Material and Methods

### 4.1. Ethics Statement

Informed consent was obtained from all participants before this study was conducted. This study and all procedures were approved by Jiaxing Municipal Center for Disease Control and Prevention (Approval code: 2022-03) and carried out in accordance with the biosafety and ethical standards of the institutional and national research committee and the relevant laws and regulations of the People’s Republic of China.

### 4.2. Specimens and Positive Controls

In total, 338 cholera-related specimens and 22 isolated bacteria strains used in this study, mainly from Jiaxing Municipal Center for Disease Control and Prevention (Jiaxing CDC), submitted by various hospitals in Jiaxing from 2013 to 2020, and partially from Zhejiang Provincial Center for Disease Control and Prevention (Zhejiang CDC). These specimens were mostly anal swabs, a small number of vomit samples, collected in transport medium and stored at −20 °C, and some environmental water samples stored in bags at 4 °C. All of them were tested by commercial real-time PCR kits or previous in-house monoplex real-time PCR assay (see below) according to the *Chinese* *Cholera Prevention* *Manual* (Sixth Edition).

Additionally, all positive standards of *Vibrio cholerae* were prepared using target DNA plasmids (*Escherichia coli* pUC57 vector) synthesized by Shanghai Sangon Biotech Co., Ltd. (Sangon) according to the reference sequences (Table 4). The nucleic acid extraction was performed with a MagMAX™-96 Viral RNA/DNA Isolation Kit (Catalog#AM1836, Ambion, Waltham, MA, USA) on a KingFisher Flex system (Thermo Fisher Scientific Inc., Waltham, MA, USA). The DNA extracts were quantified and calibrated with a NanoDrop™ 2000 microspectrophermeter (Thermo Fisher Scientific Inc., Waltham, MA, USA) and adjusted to 10^10^ copies/mL of target DNA using an EASY Dilution for Real-Time PCR (Cat#D9160A,TaKaRa) as the original quantification standards in this study. The dilutions containing 10^8^ copies/mL of DNAs were used as positive controls and 10-fold serial dilutions were used to determine the analytical sensitivity of the following assays. All of the original standards and dilutions were stored at −80 °C until analysis.

### 4.3. Bacterial Nucleic Acid Extraction

Before bacterial nucleic acid extraction, bacterial isolates and specimens were incubated in alkaline peptone water (pH 8.0) for 6 h at 36 °C, and bacteria solutions were adjusted to 0.5 MCF (McFarland concentration) with normal saline. In total, 75 μL of solution was used to extract bacterial nucleic acid using the MagMax™-96 RNA/DNA Isolation Kit on the KingFisher Flex system according to the manufacturer’s recommendation. The nucleic acid extracts were collected and stored at −80 °C before use. In addition, the *epsM*-positive control, containing 10^5^ copies/μL of DNA of *Vibrio cholerae* species-specific gene, was added to the extraction procedure in order to monitor the DNA extraction efficiency by comparing the concentrations before and after the extraction.

### 4.4. Primers and Probes

The primers and TaqMan probes used in this study are listed in Table 5. Those targeting the *epsM,* *ctxA,* *rfb-O1,* and *rfb-O139* genes for our multiplex real-time assay were newly designed by our lab and used double-quenching probes with two black-hole quenching (DBQ) groups. Others used in the previous monoplex real-time assay were consistent with the sequences and labeled fluorescence recommended in the *Cholera Prevention Manual*, targeting the *ctxA*, *rfb-O1*, and *rfb-O139* genes. All of the forward primers, reverse primers, and probes were synthesized by Shanghai Generay Biotech Co., Ltd. (Generay, Shanghai, China).

### 4.5. Previous Monoplex Real-Time Assay

The monoplex real-time PCR assay adopted in our lab previously had three panels to, respectively, detect 3 target genes, *ctxA*, *rfb-O1*, and *rfb-O139*, of *Vibrio cholerae* based on universal reagent Taq Pro HS Universal U+ Probe Master Mix (Cat# QN114, Vazyme Biotech Co., Ltd., Nanjing, China), using the primers, probes, and cycling conditions recommended in the *Cholera Prevention Manual* and the kit instructions. The final 25 μL mixture for each reaction contained 5 μL of nucleic acid extract and the optimized concentrations of the primers (-F and -R) and TaqMan probes (Table 5). The cycling condition was 37°C for 2 min, 95 °C for 30 s, and 40 cycles of 95 °C for 10 s and 60 °C for 30 s, and the assay was performed in a CFX96™ Touch Real-time PCR System (Bio-Rad Laboratories Inc., Hercules, CA, USA). The qualitative result of the assay was determined as follows: the threshold cycle value (*C_T_*) < 35, positive; no amplification or *C_T_* > 38 (considered as an invalid amplification), negative; *C_T_* between 35 and 38, reserved, if still in the range between 35 and 38 after repeat, it was considered as positive. The average *C_T_* value was determined for each standard dilution in two replicates, and the detection limit of the assay for each target was estimated from the standard curve at the cutoff point of the *C_T_* value (the value was set to 38 in this study).

### 4.6. New Multiplex Real-Time Assay

A new one-step multiplex direct real-time RT-PCR assay for 4 targets, including *epsM*, *ctxA*, *rfb-O1*, and *rfb-O139*, was developed by our lab, based on a universal reagent HiScript^®^II U^+^ One Step qRT-PCR Probe Kit (Cat# Q222-CN, Vazyme Biotech Co., Ltd., Nanjing, China), in order to simultaneously amplify and detect the DNA and RNA targets of *Vibrio cholerae*. The novelty was that the mild cell-lysing agent Triton X-100 (0.32%vol) and PCR enhancer Betaine (1 M) were added into the PCR system to release bacterial nucleic acids and enhance the PCR reaction. The final 25 μL mixture for each reaction contained 5 μL of 0.5 MCF bacteria suspension and the optimized concentrations of the primers (-F and -R) and TaqMan probes (Table 5). The real-time RT-PCR was performed in the CFX96™ Touch Real-time PCR System under the cycling condition of 50 °C for 10 min, 95 °C for 30 s, and 40 cycles of 95 °C for 2 s and 60 °C for 20 s. The qualitative result and the detection limit of the assay were determined the same as the monoplex real-time PCR assay described above.

## 5. Conclusions

The one-tube direct multiplex real-time RT-PCR assay developed by our laboratory has obvious advantages of rapidity, specificity, and sensitivity and is suitable for emergency detection during cholera epidemics. Additionally, with a high-throughput capability to process large numbers of specimens simultaneously, significantly reducing the cost, it is also suitable for mass surveillance during non-epidemic periods. Furthermore, due to the flexibility and novelty in extraction-free, direct PCR and high-speed amplification, the assay can be expanded to rapidly detect other pathogen targets to adapt to local seasonal or emerging changes in other infectious diseases.

## Figures and Tables

**Table 1:** Qualitative results of 38 specimens or strains using the two real-time assays.

Samples &. No.		Monoplex Assay			Multiplex Assay			
		*CtxA*	*Rfb-O1*	*Rfb-O139*	*EpsM*	*CtxA*	*Rfb-O1*	*Rfb-O139*
**Feces of patients** ^a^	FC1	+ ^c^	− ^c^	+	+	+	−	+
	FC2	+	−	+	+	+	−	+
	FC3	+	−	+	+	+	−	+
Anal swabs of patients ^a^	AS1	+	−	+	+	+	−	+
	AS2	+	−	+	+	+	−	+
	AS3	+	−	+	+	+	−	+
Foods from outbreaks ^a^	FD1	+	−	+	+	+	−	+
	FD2	+	−	+	+	+	−	+
	FD3	+	−	+	+	+	−	+
Waters from outbreaks ^a^	WT1	+	−	+	+	+	−	+
	WT2	+	−	+	+	+	−	+
	WT3	+	−	+	+	+	−	+
Waters from an epidemic spot in a 2013 outbreak	WA1	+	+	−	+	+	+	−
	WA2	+	+	−	+	+	+	−
	WA5	+	+	−	+	+	+	−
	WA6	+	+	−	+	+	+	−
Collected bacteria strains ^b^								
*V. cholerae* O1,Ogawa serotype	ATCC14035	+	+	−	+	+	+	−
*V. cholerae* O1,Ogawa serotype	130605	+	+	−	+	+	+	−
*V. cholerae* O1,Ogawa, *ctx*-negative	200907	−	+	−	+	−	+	−
*V. cholerae* O1,Inaba serotype	200806	+	+	−	+	+	+	-
*V. cholerae* O1,Inaba, *ctx*-negative	190625	−	+	−	+	−	+	−
*V. cholerae* O139(0511)	180511	+	−	+	+	+	−	+
*V. cholerae* O139(0618)	180618	+	−	+	+	+	−	+
*V. cholerae* O139, *ctx*-negative	200513	−	−	+	+	−	−	+
*V. cholerae* non-O1/non-O139,*ctx*-postive	190524	+	−	−	+	+	−	−
*V. cholerae* non-O1/non-O139	090717-203	−	−	−	+	−	−	−
*V. cholerae* non-O1/non-O139	090717-207	−	−	−	+	−	−	−
*V. cholerae* non-O1/non-O139	190626	−	−	−	+	−	−	−
*V. cholerae* non-O1/non-O139	160517	−	−	−	+	−	−	−
*V. cholerae* non-O1/non-O139	170626	−	−	−	+	−	−	−
*V. cholerae* non-O1/non-O139	191016	−	−	−	+	−	−	−
*Vibrio mimicus*	ATCC33653	−	−	−	−	−	−	−
*Vibrio parahaemolyticus*	ATCC33847	−	−	−	−	−	−	−
*Aeromonas hydrophila*	8315	−	−	−	−	−	−	−
*Staphylococcus aureus*	ATCC25923	−	−	−	−	−	−	−
*Escherichia coli*	ATCC25922	−	−	−	−	−	−	−
*Salmonella anatum*	ATCC50083	−	−	−	−	−	−	−
*Shigella flexneri*	ATCC51573	−	−	−	−	−	−	−
Positive Total		22	9	15	31	22	9	15

^a^ From patients and environments in a diarrhea outbreak in 2018. ^b^ Strains collected between 2013 and 2020. ^c^ Symbols: +, positive; −, negative.

**Table 2:** Consistency in the diagnostic performances of the two real-time assays.

Target	Monoplex Assay	Multiplex Assay				Accordance Rate (%)	*Kappa*	*χ2*	*p*
		+	−	Sensitivity (%)	Specificity (%)				
*V. Cholerae* ^a^	+	124	0	100.00	17.29	47.63	0.133	175.006	<0.001
	−	177	37						
*ctxA*	+	124	0	100.00	99.53	99.70	0.994	0.000	0.990
	−	1	212						
*rfb-O1*	+	79	0	100.00	97.70	98.24	0.952	4.167	<0.05
	−	6	255						
*rfb-O139*	+	46	1	97.87	99.31	99.11	0.963	0.000	0.990
	−	2	288						

^a^ Multiplex assay used the *epsM* gene as the species-specific target of *V. cholerae.* Monoplex assay used *ctxA* as the target previously.

**Table 3:** Cost-effectiveness comparison of the previous monoplex assay and new multiplex assay.

Assays	Time /Plate	Time (2 Plates)	Cost /Reaction ^a^	Cost (2 Plates) ^a^
Previous 3-panel monoplex assay				
DNA and RNA extraction	30 min	30 min	¥30.0	¥5760.0
First real-time PCR Panel 1(25 μL) for ctxA	50 min	50 min	¥10.0	¥1920.0
Vazyme qPCR reagents (UDG+)			¥4.5	
Primers (forward and reverse, 1 target)			¥0.5	
TaqMan fluorescent probe (1 target)			¥5.0	
First real-time PCR Panel 2 (25 μL) for O1 target		50 min	¥10.0	¥1920.0
First real-time PCR Panel 3 (25 μL) for O139 target		50 min	¥10.0	¥1920.0
Second real-time PCR Panel 1–3 (25 μL) for 3 targets		150 min	¥30.0	¥5760.0
Total	80 min	330 min	¥90.0	¥17,280.0
New one-tube multiplex assay				
DNA and RNA extraction	0 min	0 min	¥0.0	¥0.0
Real-time RT-PCR (25 μL volume)	40 min	80 min	¥30.0	¥5760.0
Vazyme One Step qRT-PCR reagents (UDG+)			¥8.0	
Primers (forward and reverse, 4 targets)			¥2.0	
TaqMan fluorescent probe (4 targets)			¥20.0	
Total	40 min	80 min	¥30.0	¥5760.0

^a^ Based on the purchase prices in China in 2021 and the condition in an ordinary lab with only one real-time PCR and a nucleic acid extraction instrument. These values do not include costs for common lab reagents, disposables, instruments, labor, and development.

**Table 4:** Positive standards in this study.

Positive Standards ^a^	Sequence (5′-3′)	Reference Seq ID and Position(bp) ^b^
epsM-PC	CGCAATTGGTCTCATGGATTGCGTATTTGCAAGAGCGCCAAGGGGTGAGCGTGGATGCGATTGATATTGACCGTGGTAAAGTGAACGGCGTTGTGGAAGTCAAACGTCTGCAACTGAAGC	DQ775328.1: 371–490
ctxA-PC	AGTTCATTTTGGGGTGCTTGATGAACAATTACATCGTAATAGGGGCTACAGAGATAGATATTACAGTAACTTAGATATTGCTCCAGCAGCAGATGGTTATGGATTGGCAGGTTTCCCTCCGGAGCATAGAGCTTGGAGGGAAGAGCCGTG	AB699245.1: 441–590
O1-PC	CGTTGGGAATAACTCAAGGCGATGAAGTGATTGTACCAACATTCACTTATGTTGCCTCGGTTAATACCATAGTCCAGTGTGGTGCGTTACCCGTTTTTGCTGAAATCGAAGGTGAGTCTCTACAAGTGAGCGTAGAGGAC	X59554.1: 5741–5880
O139-PC	GATCGTGCTACGATGGCGTGTTCATTAGAAGGGCGGGTTCCCTTGTTAGACCACCGCATTGCTGAGTTTGCTGCCAGTTTGCCGATCCATTTGAAATACCGAGGTGGAAAGGGAAAGTGGCTTTTACGAGAAGTACTGTATCGTTATGTACCTAAAAAAT	AB012956.1: 34141–34300

^a^ Each covers the amplified target regions of the two assays and serves as their common positive control. ^b^ Reference seq ID is the accession No. of the sequence in NCBI GenBank.

**Table 5:** Primers and probes in this study.

Target	Primer or Probe ^a^	Sequence (5′-3′)	Reference Seq and Position(bp) ^b^	Reaction Conc.(uM)
*epsM*	epsM-F	GGTCTCATGGATTGCGTATTTG	DQ775328.1:378–399	0.4
	epsM-R	GTTGCAGACGTTTGACTTCC	DQ775328.1:465–484(-)	0.4
	epsM-P ^c^	ACGGTCAATATCAATCGCATCCACGCT	DQ775328.1:418–444(-)	0.2
*ctxA*	ctxA-F	GGGTGCTTGATGAACAATTACA	AB699245.1:452–473	0.5
	ctxA-R	TTCCCTCCAAGCTCTATGC	AB699245.1:564–582(-)	0.5
	ctxA-P ^d^	ACCTGCCAATCCATAACCATCTGCTGC	AB699245.1:526–552(-)	0.25
	m-ctxA-F	CTTCCCTCCAAGCTCTATGCTC	AB699245.1:562–583(-)	0.2
	m-ctxA-R	TACATCGTAATAGGGGCTACAGAG	AB699245.1:470–493	0.2
	m-ctxA-P ^g^	ACCTGCCAATCCATAACCATCTGCTGCTG	AB699245.1:524–552(-)	0.2
*rfb-O1*	O1-F	CAAGGCGATGAAGTGATTGTA	X59554.1:5755–5775	0.4
	O1-R	CGCTCACTTGTAGAGACTCA	X59554.1:5853–5872(-)	0.4
	O1-P ^e^	ACGGGTAACGCACCACACTGGACTATG	X59554.1:5808–5834(-)	0.2
	m-O1-F	GGAATAACTCAAGGCGATGAAGTG	X59554.1:5746–5769	0.2
	m-O1-R	TAGAGACTCACCTTCGATTTCAGC	X59554.1:5839–5862(-)	0.2
	m-O1-P ^g^	AAACGGGTAACGCACCACACTGGACT	X59554.1:5811–5836(-)	0.2
*rfb-O139*	O139-F	GGTACATAACGATACAGTACTTCTC	AB012956.1:34268–34292(-)	0.5
	O139-R	CGATGGCGTGTTCATTAGA	AB012956.1:34151–34169	0.5
	O139-P ^f^	CCTTGTTAGACCACCGCATTGCTGAGT	AB012956.1:34181–34207	0.25
	m-O139-F	CGATGGCGTGTTCATTAGAAGG	AB012956.1:34151–34172	0.2
	m-O139-R	TCCCTTTCCACCTCGGTATTTC	AB012956.1:34233–34252(-)	0.2
	m-O139-P ^g^	CGGCAAACTGGCAGCAAACTCAGCA	AB012956.1:34200–34224(-)	0.2

^a^ -F, -R, and -P represents the forward primer, reverse primer, and TaqMan probe. Those with the m- prefix were only used for the monoplex real-time assay. ^b^ Reference seq ID is the accession No. of the sequence in NCBI GenBank. The sequences with minus marks are antisense. ^c,d,e,f^ Probes labeled, respectively, with FAM-DBQ1, HEX-DBQ1, ROX-DBQ2, and CY5-DBQ2 (5′-3′) for multiplex real-time assay. ^g^ Probes labeled with FAM-BHQ1 used for monoplex real-time assay.

## Data Availability

The data presented in this study are available on request from the corresponding author. The data are not publicly available due to relative patent applications.
